# Rush Medical College

**Published:** 1865-02

**Authors:** 


### EDITORIAL AND MISCELLANEOUS,

### RUSH MEDICAL COLLEGE.

The Commencement Exercises of the Twenty-Second Annual Session of Lectures in Rush Medical College, were held on the evening of the 25th of January, the graduating class numbering 108. The address was given by Prof. Carr, of the Wisconsin University, who, during the temporary absence of Prof. Blaney, in the Medical Service of the Army, has given the course on Chemistry in a manner most acceptable to the class, and to elicit from the Trustees and the Faculty of the College the highest commendation. Of the rare excellence of the Address, its correct teaching, scholastic diction, depth of thought, and beauty of illustration, we need not speak, as we hope to present it to our readers in the pages of the *Journal.* The session thus happily brought to a close, was commenced under the brightest auspices, which, in its progress, never for a moment were overcast.

The Faculty labored, as they always have, in a spirit of perfect harmony, and with scrupulous fidelity to what they conceived to be for the best interest of their class. The lectures were given in their regular order, without change, interruption, or derangement. Of the class—the largest the College ever welcomed to its teachings—we speak with pleasure, not unmingled with pride. A somewhat extensive observation has not brought to our notice one better endowed with native capacity or possessed of higher scientific attainments than this. As a whole, they were men of superior character—their deportmen on every occasion received from all the highest praise, and they evinced an interest in the lectures that made them attentive and constant listeners. It is upon its Alumni that the reputation, success, and usefulness of the School rests, as upon a sure foundation, and the final examination of the graduating class made the Faculty incorporate them into this body without fear that its reputation would suffer thereby. All were blessed with a good degree of health, and the course was in every way satisfactory to both teachers and pupils.

The following named gentlemen received the Degree :—

| NAMES. | SUBJECT OF THESIS. |
|---|---|
| W. R. Adair, | Gunshot Wounds. |
| J. Madison C. Adams, | Milk Sickness. |
| Henry Allen, | Attraction as Applied to Animal Organism. |
| R. M. Allen, | Milk Sickness. |
| W. C. Baird, | Pneumonia. |
| Braxton Baker, | Digestion. |
| Zopher Ball, | Fractures. |
| John Becker, | Uterine Hemorrhage. |
| Newton Baker, | Pneumonia. |
| C. R. Blackall, | Mental Complications in Disease. |
| E. J. Bond, | Continued Fever. |
| D. W. Bosley, | Etiology. |
| W. E. Bowman, | Shot through the Heart. |
| James G. Boardman, | Hospital Gangrene. |
| J. W. Brown, | Gun-shot Wounds. |
| W. H. Bright, | Diphtheria. |
| J. G. Blanchard, | Gonorrhœa. |
| C. H. Brunk, | Typhoid Fever. |
| C. H. Carlisle, | Bilious Fever. |
| E. P. Catlin, | Variola. |
| W. E. Chamberlin, | Scarlatina. |
| H. F. Chesbrough, | Pain. |
| Frederick Cole, | The Rational Physician. |
| Samuel Cole, Jr., | Varicose Veins. |
| H. N. Clark, | Pneumonia. |
| J. L. Congdon, | |
| J. Cooper, | Conjunctivitis. |
| John Cotton, | Camp Diarrhœa. |
| Clinton Cushing, | The Bile. |
| M. Morton Dowler, Jr., | Osteo-Genesis. |
| A. J. Darrah, | Auscultation. |
| S. A. Davison, | Inflammation. |
| S. W. Dodd, | Etiology. |
| A. C. Douglass, | Enteric Fever. |
| A. S. Ehle | Cynanche Trachealis. |
| Andrew J. Eidson | Opium. |

| NAMES. | SUBJECT OF THESIS. |
|---|---|
| Samuel S. Elder | Typhoid Fever. |
| Samuel T. Ferguson | " " |
| S. A. Ferrin | Pseudo-Membranous Croup. |
| Henry A. Folger | Therapeutical Agents. |
| O. D. Ford | Pernicious Fever. |
| J. H. Foster | Conservative Medicine. |
| Samuel Galloway | Dysentery. |
| H. T. Godfrey | Small Pox. |
| R. Romanta Gaskill | Rubeola. |
| J. Thomas Hale | Typhoid Fever. |
| J. M. Hurrah | Criminal Abortion. |
| Thos. C. Hance | Typhoid Fever. |
| A. P. Herndon | Intermittent Fever. |
| Wm. H. Hess | Causes, Symp. and Treatment of Inflmmation. |
| Smith H. Hess | Scarlatina. |
| J. W. Herdman | Inflammation. |
| Francis M. Hiett | Intermittent Fever. |
| H. Edward Horton | Blood in Disease. |
| Geo. W. James | Phthisis. |
| Merritt S. Jones | Erysipelas. |
| David R. Johnston | The Prostrate Gland. |
| Charles Kerr | Cholera Infantum. |
| G. F. Keiper | Placenta Prævia. |
| W. J. Kelsey | Necrosis. |
| John L. Kite | Hospitals. |
| Chas. Ed. Kuester | The Spleen. |
| C. E. Lamon | Milk Sickness. |
| J. H. Leal | Prophylaxis. |
| Josiah Lee | Erysipelas. |
| C. J. Lewis | " |
| A. W. Lueck | Respiration. |
| Carl J. Lucas | Sterility. |
| W. B. Lyons | Spotted Fever. |
| Isaac L. Mahan | Typhoid Fever. |
| J. G. Meachem, Jr | Albuminuria. |
| L. B. Morrow | Natural Labor. |
| William A. Morse | Camp Diarrhœa. |
| G. D. Maxon | Cerebritis. |
| William M. Newell | Man as a Machine. |
| M. W. Nesmeth | Intermittent Fever. |
| Joseph Otto | Blood. |
| William P. Penfield | Pneumonia. |
| John W. Powell | Gonorhœa. |
| Joseph L. Prentiss | Blood. |

| NAMES. | SUBJECT OF THESIS. |
|---|---|
| G. W. Priest | Evidences of Gestation. |
| Charles H. Quinlan | Preservation of the Teeth. |
| Lafayette Redmon | Pneumonia. |
| A. J. Rodman | Potassium. |
| C. B. Reed | Diphtheria. |
| Flavel Shurtleff | Diabetes. |
| J. L. Shepard | Variola. |
| Emery Sherman | Hygeine. |
| Ashbury E. Smith | Diphtheria. |
| W. H. H. Smith | Typhoid Fever. |
| M. S. Stahl | Digestion. |
| G. A. Stevenson | Management of Natural Labor. |
| D. Hedrick Stratton | Toxæmia. |
| G. C. Smythe | Military Hospitals. |
| J. L. Trousdale | Progress of Medical Science. |
| John W. Trueworthy | Delirium Tremens. |
| Henry Van Buren | Dropsy. |
| G. W. Van Zant | Inflammation. |
| Theodore Wild | Vis Medicatrix Natura. |
| Joseph H. Wilson | Management of Natural Labor. |
| Horatio B. Withers | The Accouchuer. |
| George Worsely | Typhoid Pneumonia. |
| O. P. B. Wright | The Circulatory System. |
| Charles Young | Fever. |

The *ad eundum* degree was conferred on the following named gentlemen:

Martin Baker, M. D., California.
D. W. C. Denny, M. D., Indiana.
W. H. Dubler, M. D., Illinois.
N. Wright, M. D., Illinois.

